# Identification of different species of *Zanthoxyli Pericarpium* based on convolution neural network

**DOI:** 10.1371/journal.pone.0230287

**Published:** 2020-04-13

**Authors:** Chaoqun Tan, Chong Wu, Yongliang Huang, Chunjie Wu, Hu Chen

**Affiliations:** 1 National Key Laboratory of Fundamental Science on Synthetic Vision, College of Computer Science, Sichuan University, Chengdu, China; 2 College of Pharmacy, Chengdu University of Traditional Chinese Medicine, Chengdu, China; 3 Affiliated Hospital of Chengdu University of Traditional Chinese Medicine, Chengdu, China; Newcastle University, UNITED KINGDOM

## Abstract

*Zanthoxyli Pericarpium* (ZP) are the dried ripe peel of *Zanthoxylum schinifolium* Sieb. et Zucc (ZC) or *Zanthoxylum bungeanum* Maxim (ZB). It has wide range of uses both medicine and food, and favorable market value. The diverse specifications of components of ZP is exceptional, and the common aims of adulteration for economic profit is conducted. In this work, a novel method for the identification different species of ZP is proposed using convolutional neural networks (CNNs). The data used for the experiment is 5 classes obtained from camera and mobile phones. Firstly, the data considering 2 categories are trained to detect the labels by YOLO. Then, the multiple deep learning including VGG, ResNet, Inception v4, and DenseNet are introduced to identify the different species of ZP (HZB, DZB, OZB, ZA and JZC). In order to assess the performance of CNNs, compared with two traditional identification models including Support Vector Machines (SVM) and Back Propagation (BP). The experimental results demonstrate that the CNN model have a better performance to identify different species of ZP and the highest identification accuracy is 99.35%. The present study is proved to be a useful strategy for the discrimination of different traditional Chinese medicines (TCMs).

## Introduction

*Zanthoxyli Pericarpium* (ZP) is the dried ripe peel of *Zanthoxylum schinifolium* Sieb. et Zucc (ZC) or *Zanthoxylum bungeanum* Maxim (ZB), [1] has been traditionally used for the therapy of pathogenic wind, diarrhoea, epigastric pain, pruritus, fungal infection, eczema, and dysentery.[2,3] In addition, the fruit of ZP are widely used for preparation of other agents, such as insecticides, air fresheners, pesticides, and drugs. [4,5] Simultaneously, ZP is distributed in more than 20 provinces including Sichuan. [6] Sichuan-ZP are also a popular food additive and widely used in cooking with the history of more than 1000 years for both medicinal and economic values.[7] ZB have mainly included Hanyuan ZB (HZB), Derong ZB (DZB), and other Sichuan ZB (OZB). ZC are included *Zanthoxylum armatum* (ZA) and Jinyang ZC (JZC) in this paper. Among Sichuan-ZB, Hanyuan *Zanthoxylum bungeanum* Maxim (HZB) are called “Gongjiao” in Chinese, [8] which have the great demands and high values. It has been reported that HZB compared with other Sichuan-ZB are highest content of amide and Volatile oil. [9] However, it is often cheated with other ZB and confusion of species in the market.[10] Therefore, it plays an important role for the accurate discrimination of different ZP in suitable methods.

The methods of biological evaluation and physicochemical identification are the most familiar to the discrimination of ZP. [11,12] However, the mentioned approaches have difficulties in reflecting the overall quality of ZP, demanding for specific references and being time-consuming. [13] It is reported that the external properties including color, odor, and taste are closely related to the inherent quality of ZP. [14] And the intelligent sensory technology are combined with machine learning algorithms to use for the identification of ZP. Wu *et al*. [15,16] have used electronic nose and computer vision to obtain the odor and color features, the BP and SVM are adopted for the identification and ZP. Hussein *et al*. [17] have applied Discriminant Analysis, Random Forest and SVM to identify the families Annonaceae, Euphorbiaceae and Dipterocarpaceae. The results have shown the feasibility of using extracted traits for the identification of Family Dipterocarpaceae and Euphorbiaceae, but not yield good for Annonaceae family. Tao *et al*. [18–20] have proposed to obtain various different texture features to reflect the herbal information, then used machine learning algorithms to recognize 18 herbal medicine categories. These methods have effectively identified different species. Nevertheless, the features extracted from gradient, color, texture and shape by hand-crafted directly can be easily influenced and be changed by external environment, thus the identification accuracy is affected. [21,22] In addition, the above low-level features cannot adequately describe the characteristics of discriminators, and the traditional machine learning identification models can’t discriminate accurately for practical applications with complex backgrounds. Consequently, it is pressing to need a novel method for identifying the different species of ZP.

Advances in artificial intelligence, image processing and pattern recognition can expand and improve the practice of TCM-technology. CNNs have attracted a lot of attention with the aim to develop a quick, automatic and accurate system for image identification.[23] In particular, CNNs have the powerful learning ability and expression capability of efficient feature, and it can capture information from the image by extracting from low-level features to high-level semantics.[24–26] The CNNs models have been widely used in agriculture, medical care, education, energy, industrial inspection and other fields. Xue *et al*. [27] have proposed a fast and easy method for predicting agricultural waste compost maturity by analyzing images of different composting stages, and it achieves accuracy of 99.7%, 99.4%, 99.7% and 99.5% on the 4 test sets, respectively. Park *et al*. [28] have proposed a deep learning-based player evaluation model to analyze the positive or negative effect for baseball league. Batchuluun *et al*. [29] have used CNNs to identify the body-movement-based human. Hansen *et al*. [30] have achieved the individual pig recognition with accuracy rates of 96.7%. Fricker *et al*. [31] have proposed CNNs for the identification of tree species in mixed-conifer forest from hyperspectral imagery, the results demonstrated that the method can accurately identify tree species and predict their distribution. Altuntaş *et al*. [32] have used CNNs to recognize haploid and diploid maize seeds automatically and Zhang *et al*. [33] have proposed a global pooling dilated CNN to identify six common cucumber plant disease.

There have been no reports on identification modeling of ZP based on CNNs. Due to the visually importance of exterior for the identification of ZP category, the ZP images can have been used to research the discrimination of ZP by machine learning algorithms. Therefore, the novel method is proposed and the model is built by 2 sections. The idea of YOLO is to detect the labels of images, and the multiple deep learning are used to identify ZP. The model aims to develop a rapid and accuracy strategy for discriminating the different species of ZP. It can been effectively used for the classification of ZP in different background and environment. This is a creative combination and significant practice of TCM-technology.

## Materials and methods

### Data acquisition

The samples are provided by Sichuan auxiliary Pharmaceutical Co., Ltd. (Chengdu, China). A DSLR camera (Canon EOS 60D) has been used to capture image for resolution of 5184 *3456 pixels in the visible spectrum with consistent white balancing, and exposure settings. And it has a good performance to capture images. The clear and stable dataset can be a better anti-interference effect. Environmental conditions of shooting scene have an influence on the result to a large extent. Therefore, the different types of mobile phone are also used to take images to enrich the diversity of training data in different lighting environments.

**Fig 1:**
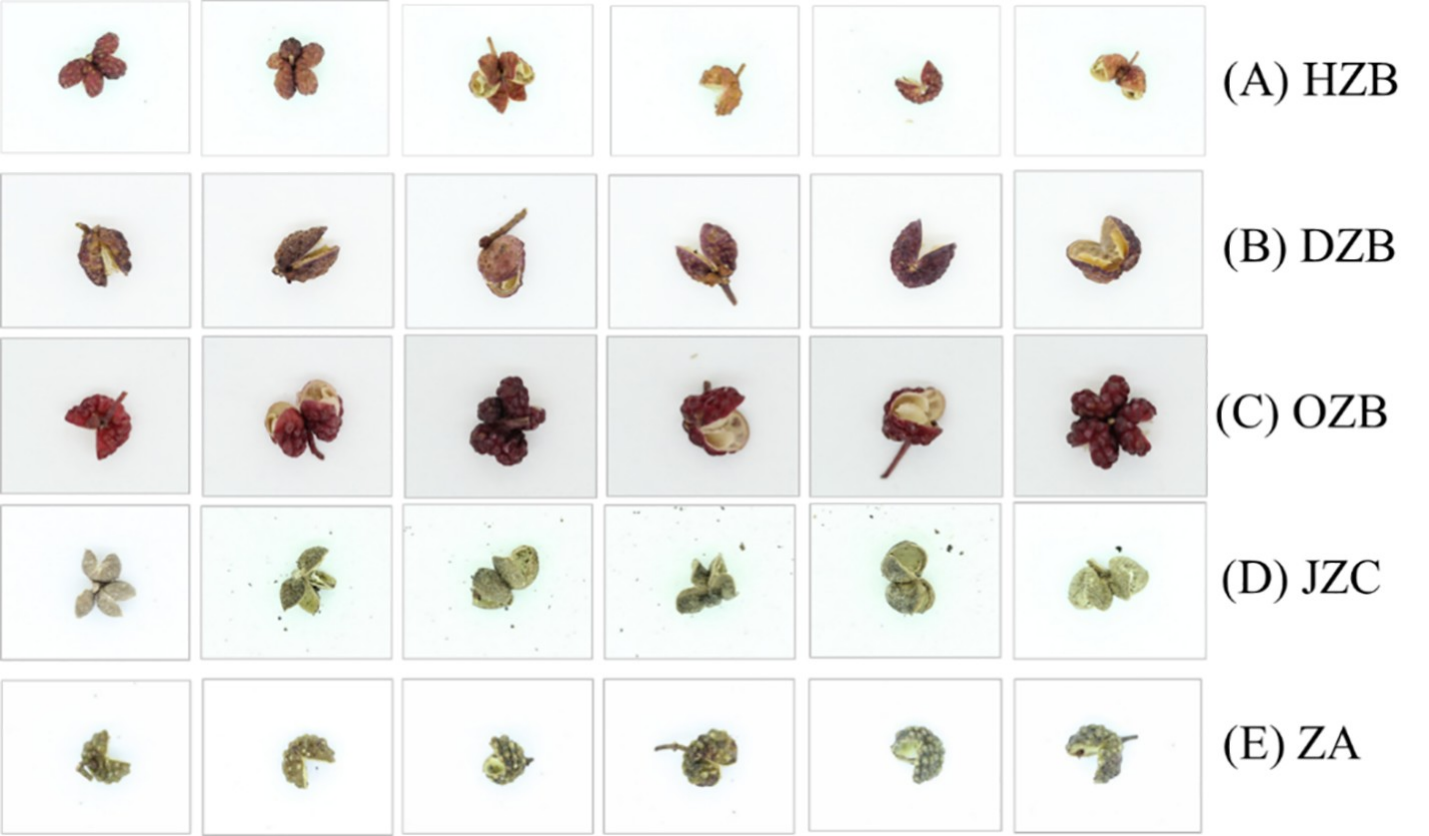
The different species of ZP image.

The dataset is referred to ZPD. The part of which is shown in Fig 1, and the number of different species ZP is listed in Table 1. It is composed of 65000.

**Table 1:** The number of images for different species ZP.

Different species of ZP	Number
OZB	14000
JZC	14000
ZA	14000
DZB	13000
HZB	10000

### State-of-the-art learning classifiers

#### Traditional identification model

The identification methods for herbal plants based on image processing have required under specific conditions. The features are extracted by hand-crafted, and the color feature is expressed by HSV, the shape feature is indicated by Hu invariant moment (HU), the texture feature is denoted by Gray-level co-occurrence matrix (GLCM). [34] The parameters must be readjusted to deal with different lightning conditions and diverse background. The identification process is shown as S1 Fig.

Firstly, all images are preprocessed by grayscale and binary processing. Then, the features of color, shape and texture have been extracted. Finally, the SVM or BP has been used to train. The training set(*x_i_,y_i_*), which *x_i_* are input, *y_i_* ∈ {1, 2 … k} are the corresponding output. The SVM regression function [35] is established as follows:

f(x)=wφ(x)+b (1)

The *w* and *b* are weight and bias vectors, respectively. The classifier has been trained the smallest training error to maximize the geometric interval. Therefore, it is necessary to find the minimum *w* to maximize the geometric distance. The slack parameter *ξ* and the penalty factor *C* are introduced to reduce the loss. The function has been converted to Eq 2 [36]:

$$\mathrm{Min}\;\frac{1}{2}{\left\|w\right\|}^{2}+C\sum_{i=1}^{n}\xi_{i}$$ (2)

$$\mathrm{Subject}\;\mathrm{to}\;y_{i}[(wx_{i})+b]\geq 1-\xi_{i}\;\xi \geq 0$$ (3)

The SVM has unique advantages in solving nonlinear pattern recognition problems. Thereby, it is a good choice for the identification of TCM.

CNN is the type of multi-layer network learning algorithm, which usually contains multiple convolutional layers, batch normalization layers, and fully connected layers. [37] The general CNN architecture is shown as S2 Fig.

#### VGG

VGG is one of common CNN, which is used 3×3 convolutional layers stacked on top of each other to increase depth. On the one hand, it can reduce the parameters. On the other hand, it is equivalent to more nonlinear mapping, which can increase the fitting ability of the network. Additionally, fully-connected layers is connected to extract high-level features. The results of fully-connected are transferred to output layer. The output is also a vector of numbers. [38] Furthermore, a pre-trained model with weights from ImageNet was used. [38] In this work, the learning rate is set to 0.0001 and the value of dropout is 0.5. And the network architecture parameters of VGG16 is shown in S1 Table.

#### ResNet

In order to solve the inability of multiple non-linear layers to learn identity mappings and degradation problem, the ResNet is proposed. ResNet is the architecture that relies on many stacked residual units, which are the set of building blocks used to construct the network. The residual units are made of convolution, pooling, and Relu layers. The accuracy can be improved by updating the residual module to use identity mappings. [39] In this paper, the ResNet101 is used to train and the network architecture parameters of ResNet101 is shown in S1 Table.

#### Inception v4

The Inception module is made up a pooling layer and convolution layers stacked together. [40] The convolutions are of varied sizes of 1×1, 3×3 and 5×5. Another salient feature of the Inception module is the use of bottleneck layer which is a 1×1 convolutions. [40] The bottleneck layer helps in reduction of computation requirements. Additionally, the pooling layer is used for dimension reduction within the module. Inception v4 replaces the filter concatenation stage of the Inception architecture with residual connections. [40] In this paper, the initial learning rate of Inceptionv4 is set to 0.0001 and the value of dropout is 0.2. The network architecture parameters of Inception v4 is shown in S1 Table.

#### DenseNet

DenseNet was proposed in 2016. [41] All layers are connected directly with each other in a feed-forward manner, the maximum information flow between layers can be obtained. During training, the feature maps are used as inputs and its own feature maps are used as inputs into all subsequent layers. This method has the advantages of reducing number of parameters, increasing transmission of features and alleviating the problem of vanishing-gradient. [41] For this task of identification, the train steps of DenseNet are similar to ResNet. In this work, the DenseNet121 is selected to train all data and the initial learning rate is set to 0.0001, the value of dropout is 0.2. The network architecture parameters of DenseNet121 is shown in S1 Table.

### Images detection

#### Images labeling

The irrelevant images detection could distort the final results. Therefore, all images have been manually labeled and preprocessed the location of target. The image tags are referred to the coordination of X-axis, Y-axis, the width and length. And the images are filtered to delete the low clarity.

#### Image detection

**Fig 2:**
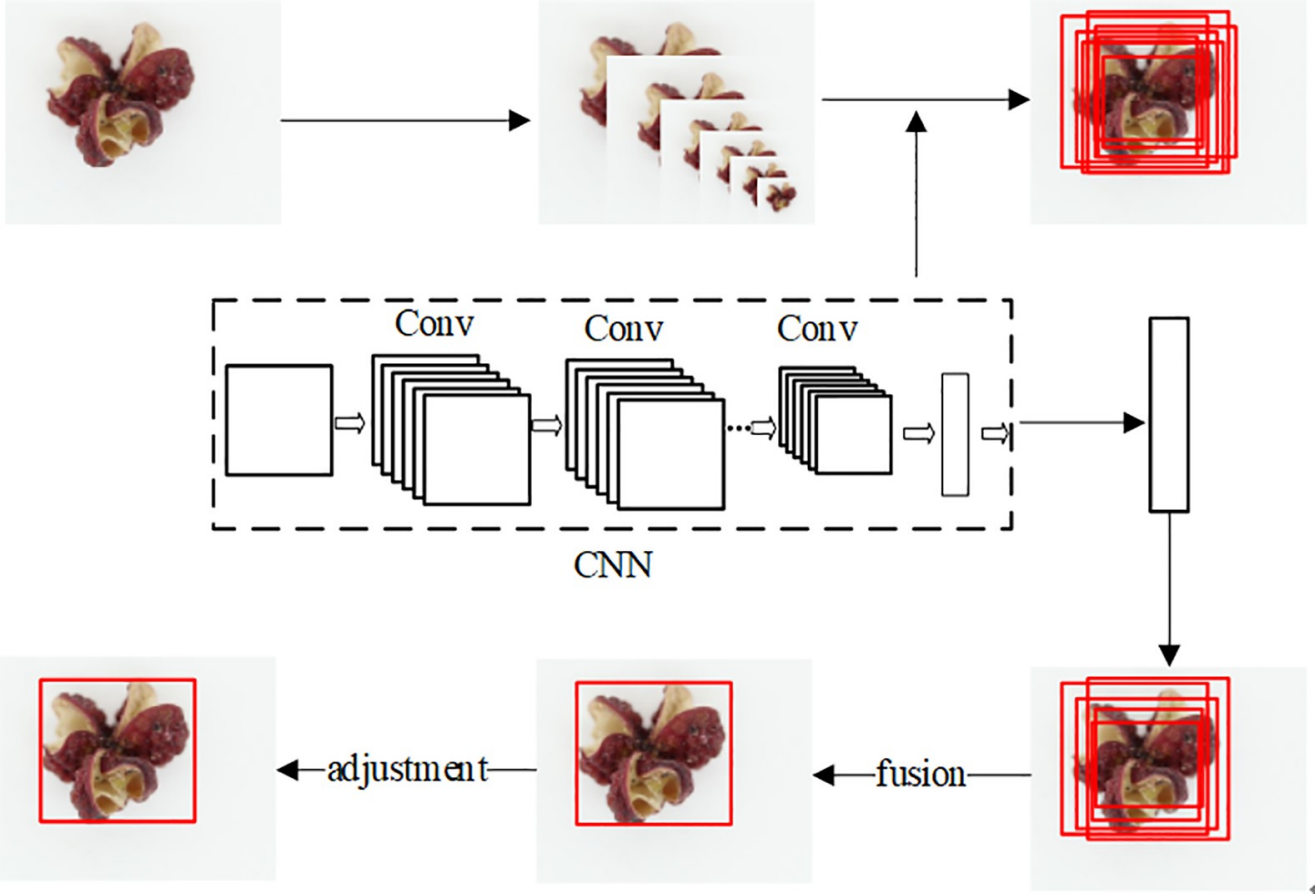
The processes of images detection.

YOLO is proposed to detect the labels for all images. This approach divides the input image into sub-regions and predicts multiple bounding boxes with their class probabilities for each region. Two bounding boxes with their confidence scores are utilized to represent each grid cell. The illustration of experimental processes is shown in Fig 2. In the detection of ZP, the best predicted labels among the inner and outer boundary of object has been gotten. It is able to detect the presence of ZP and classify for images.

The training data are prepared with labels and class label of the masses. Then the path of training and testing files are modified. The multiple bounding boxes are predicted for each region, which are merged and adjusted. The bounding box with the highest confidence score is selected as the label. Therefore, the goals of preprocessing is ensure that each image can obtain the label. According to the computed relationships of different parameter, the value of filters has been set 35.

### Image classification

#### Image preprocessing

According to the final label of the previous step, the 5 categories of images are cropped by the result of detection. In order to reduce the dimension of data and keep the details of the input, the images are preprocessed into the corresponding format required by applying the CNN.

#### Image classification

The parameter can be changed to train model. These neurons respectively correspond to a local area in image, and it is used to extract the feature of region.

### The building of recognition model

#### Architecture of the recognition model

**Fig 3:**
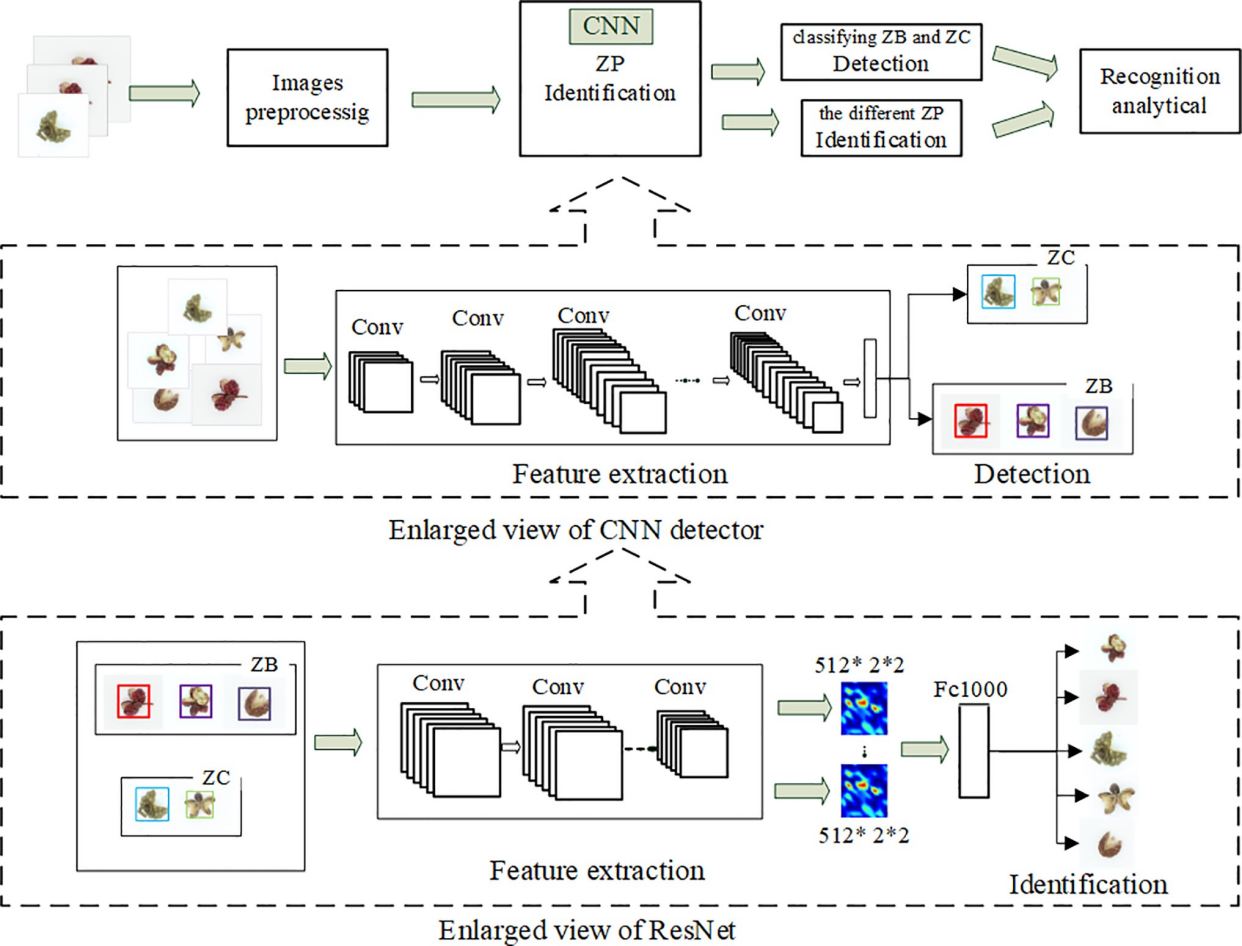
Overview of the recognition of ZP analysis procedure.

In this work, the CNN architecture including YOLO and ResNet is applied for the recognition of ZP, which is shown in Fig 3. The brief mention of relevant details regarding the implementation of the method is revealed. The proposed process is essentially consisted of two parts. The YOLO is extracted for generating a list of region proposals and detected the labels. Then, the ResNet is used to train the identification of ZP. And the remaining data are used to test the accuracy of model.

#### Train and test phase

The steps of training and testing mode are shown in S3 Fig. The training data combining original images with labels are used to train detection model, then the preprocessed images are made into final model S3(A) Fig. After the final model is trained, testing images are used to evaluate the performance of final model S3(B) Fig.

## Results

### The detection of ZC and ZB

The ZPD is divided ZC and ZB, which are processed for the specified format as input, and the fully connect layers to achieve the detection. An input of model is divided into 416*416 non-overlapped grid cells by YOLO v2. The main role of using convolutional filters is to extract different features from the images and generate the feature maps. Thus, each grid cell is responsible to detect the potential mass belonging to the cell.

The crucial parameters of Network are shown in S2 Table. According to a batch size of 64 and learning rate of 0.0001 are utilized, and the classes is set to 2 to train. Finally, the tensor of prediction with size of 35 is generated, the size of 13×13 is chosen and given the best performance compared to other sizes. The bounding box with the highest confidence score is selected as the label. Thus, the output of the YOLO represents a matrix with size of 13×13×35.

**Fig 4:**
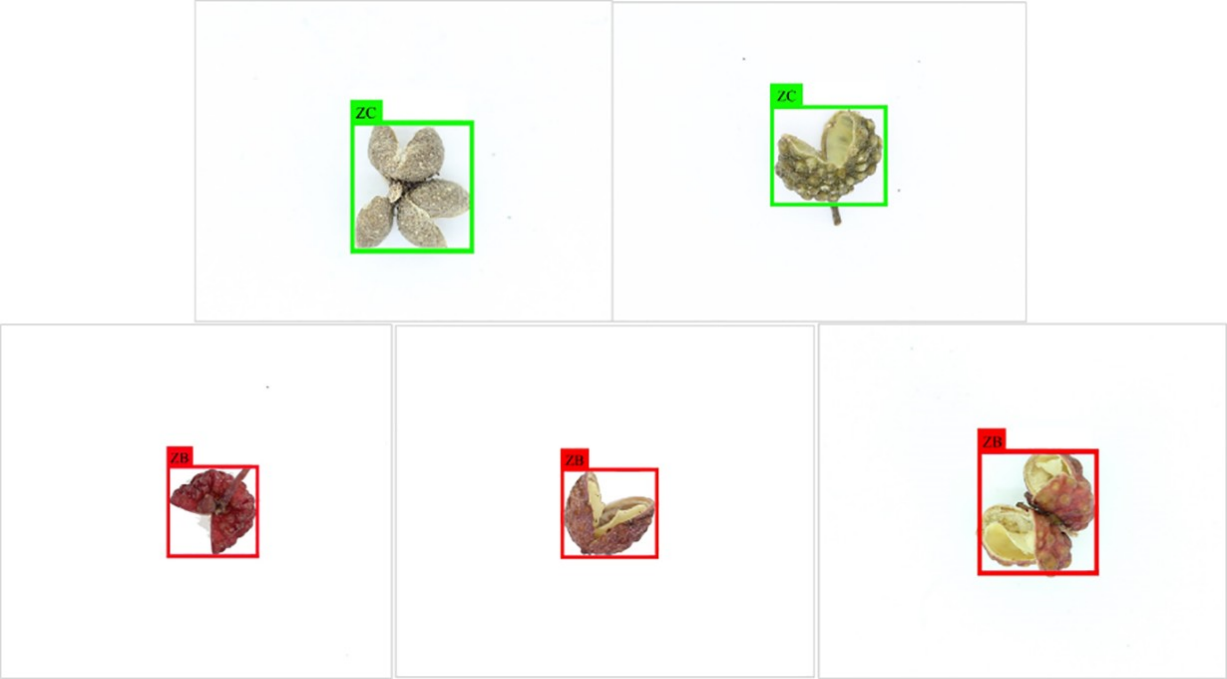
The result of images detection.

The result of detection is shown in Fig 4. The original images are fed into detection network, and the detected results output with classification,and the label of ZP. The bounding boxes are labeled and the ZB and ZC are classified. Hence, not only obtaining the label of ZP, but it is preferable to have a good performance to distinguish between ZC and ZB.

### The identification of ZP

#### Evaluation of identification performance

The crucial parameter of network structure is shown in S3 Table. The weight decay of 0.0005, step size of 10000 and gamma of 0.1 are set to train. The value of max_iter training is set to 500000. The analysis snapshot is obtained by each 10000. The learning rate is one of important parameters for the accuracy. According to the experiment, the two learning rates including 0.001 and 0.0001 are used in training. It could be summarized that no matter how the number of training set have changes, the decrease of learning rate will result in an obvious improvement for the identification precision. Thus, the highest precision appears which the learning rate of 0.0001 is chosen.

**Fig 5:**
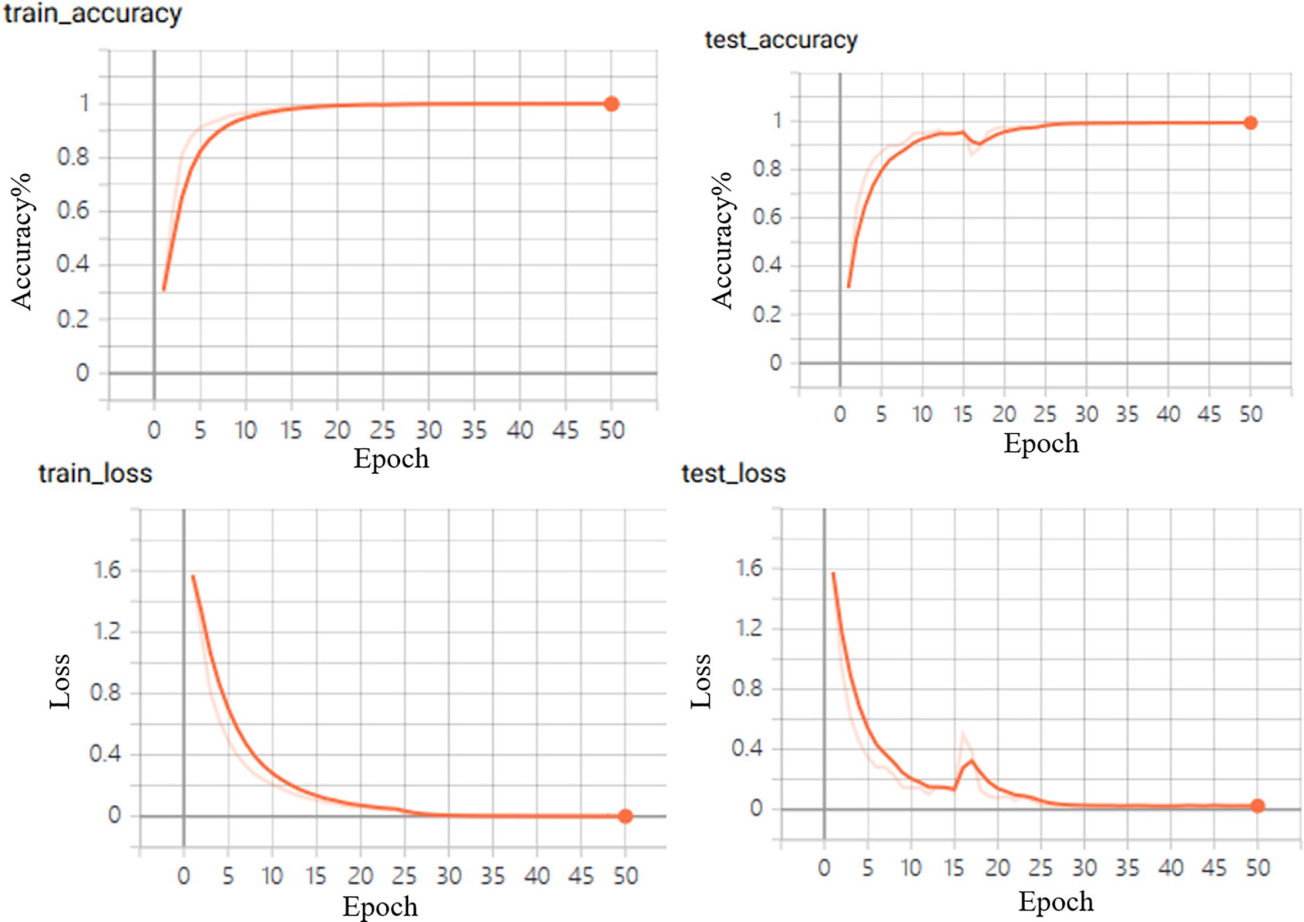
ResNet net, the accuracy and loss of the model is shown.

The ResNet is applied to achieve the identification. The data of ZB and ZC, which are labeled of 5 kinds of ZP included OZB, JZC, ZA, DZB, and HZB and numbered 0–4. All data which have been re-sized into 224×224 and combined into an image of three channels. The 5 outputs are contained in final classification layer. The accuracy and loss have been calculated, and the results are shown in Fig 5. The model have accuracy of 99.35%.

**Fig 6:**
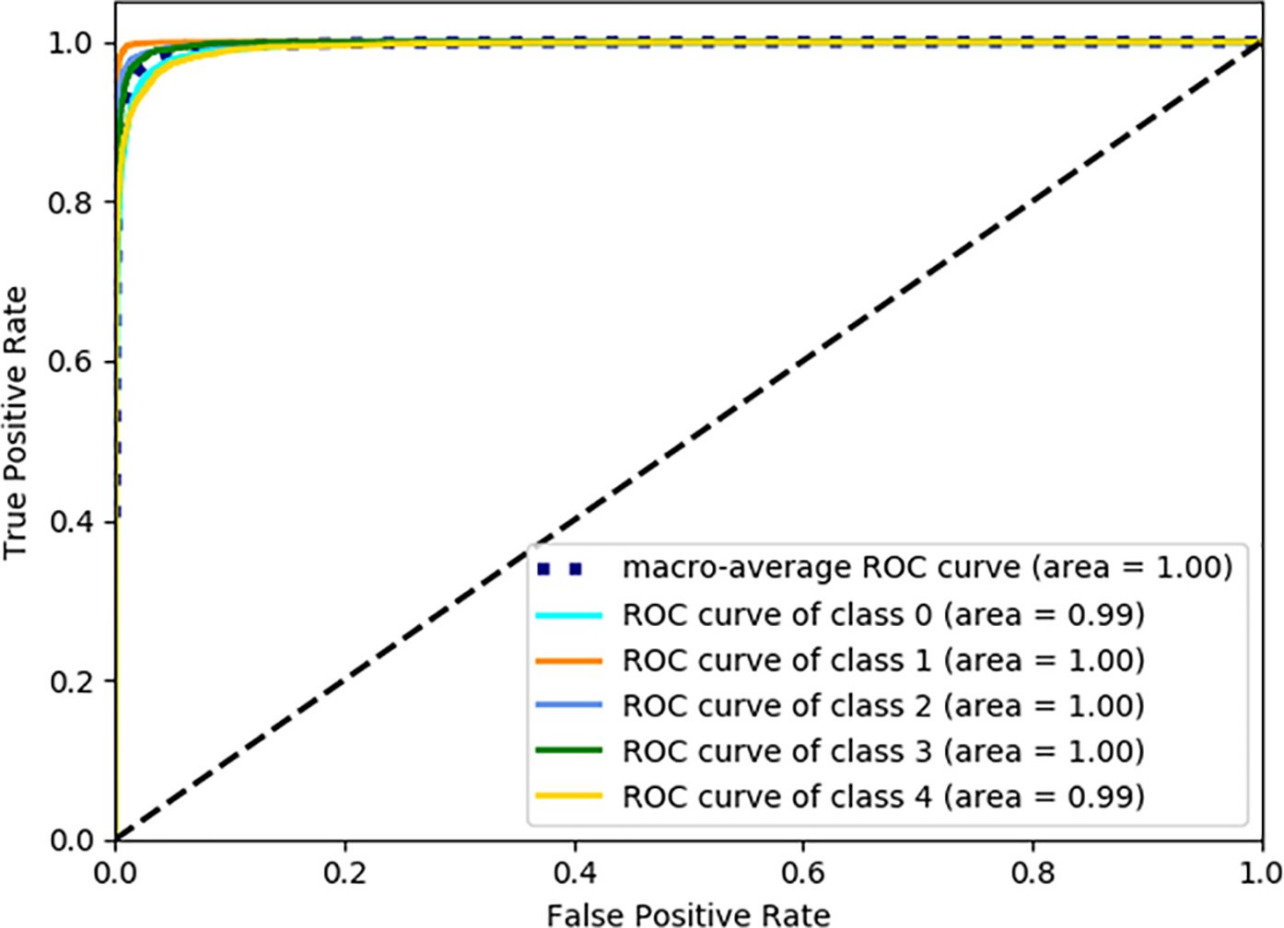
ROC curves of various classes. The “area” is the area under the TPR-FPR curve of each class.

In addition, in order to evaluate the performance of CNN model, the area under the Receiver Operating Characteristic (AUC-ROC) curve is calculated. The ROC curve plots the difference between the True Positive Rate (TPR) and False Positive Rate (FPR). The range of the FPR and TPR is between 0 and 1. [42] The TPR and FPR metrics table is shown in S4 Table and the function are calculated:

$$TPR=\frac{TP}{TP+FN},\;FPR=\frac{FP}{TP+TN}$$

where TN is the number of negatively rejected cases (True Negative); TP is the number of positively classified cases (True Positive); FN indicates the number of cases that are positive but negatively detected (False Negative); FP indicates the number of cases which are negative but positively detected(False Positive). The ROC curve is depicted in Fig 6.

The ROC curve is another evaluation indexes of performance for reflecting the effect of threshold. The area under the curve is called AUC, which is used to convey the accuracy. The higher of the AUC value, the larger of the area, and the higher of identification accuracy. Above the Fig 6, the results have indicated the ResNet has a good performance to classify.

#### Visual analysis of the CNN layers

**Fig 7:**
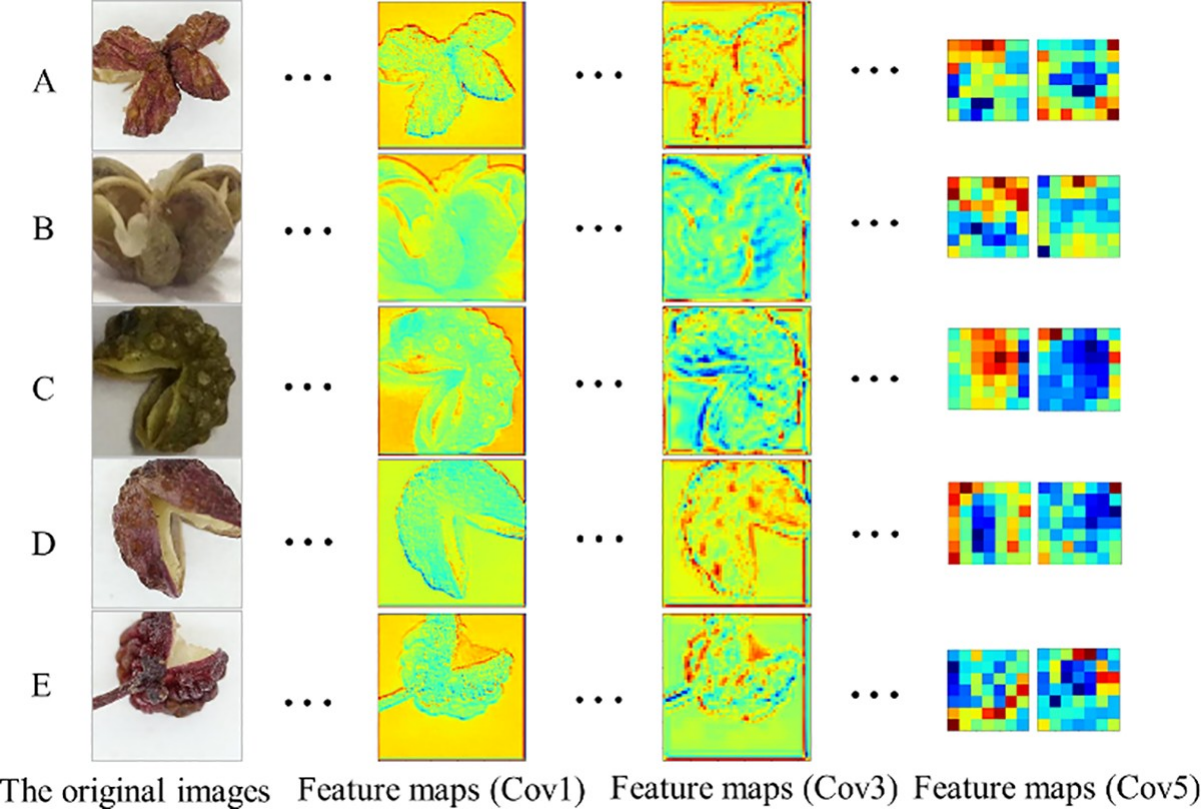
The corresponding feature maps of Conv1 and Conv5 for ZP.

The advantage of CNN is that high-level semantics features can be extracted automatically from data avoid extracting by hand-crafted design. The visualization of feature maps is analyzed to discover which features the CNN learns and trains, how activation works for different layers. The feature maps of CNN different layers is shown in Fig 7.

The outputs of network are corresponding to the features learned and trained by convolutional layers. As we can found, the earlier convolutional layers learn low-level features like color, texture and shape, which more abstract feature can be learned and represented by the deeper layers like profiles. If data with the similar species are fed to the network, the CNN will learn the mimic features and then identify the different species of ZP.

#### Comparison with multiple CNNs

**Fig 8:**
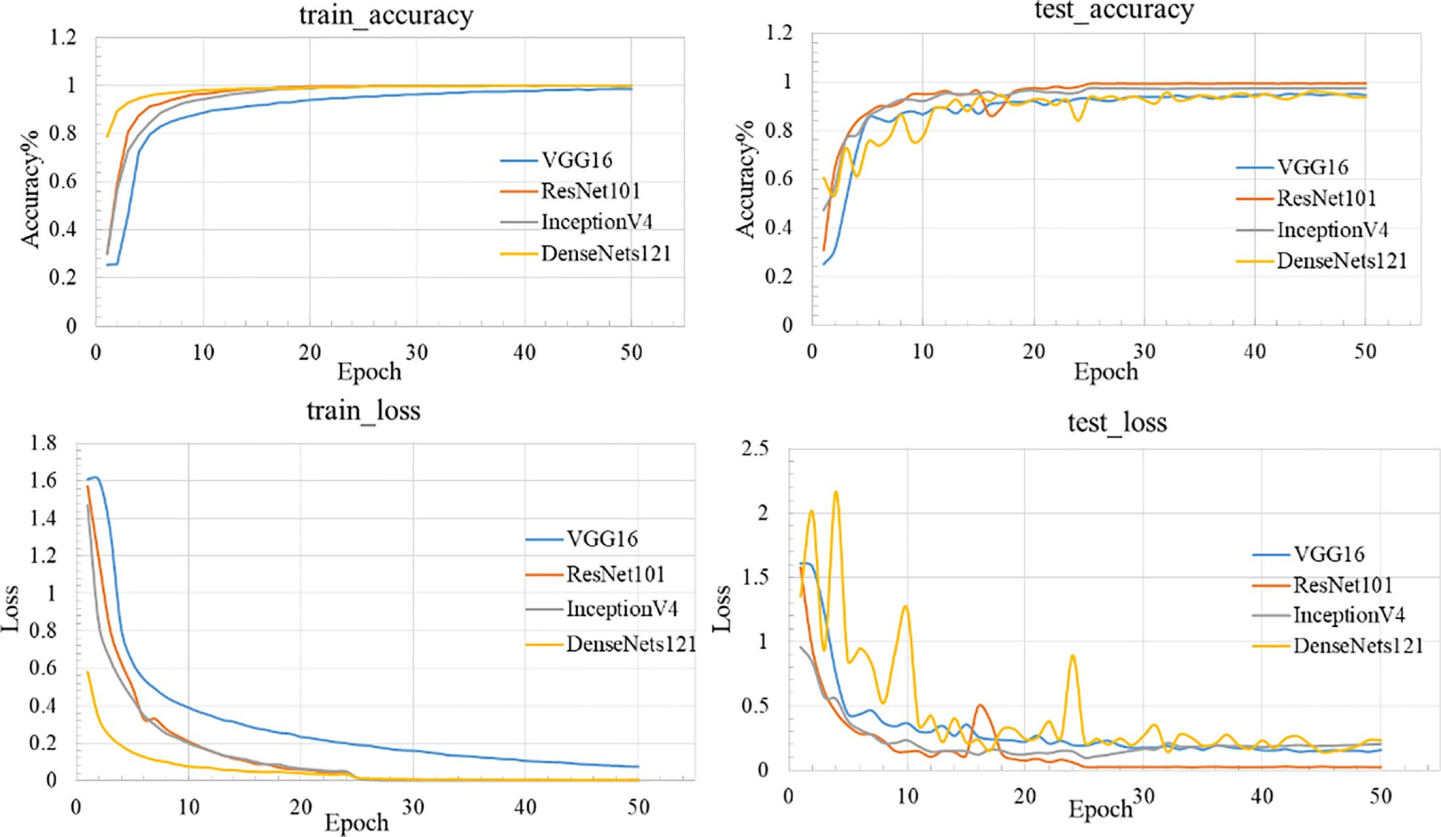
The comparison results of different four networks.

In this paper, the assessment of the identification of different species ZP are done and compared with state-of-the-art CNNs including VGG16, DenseNets121 layers, and InceptionV4. And comparison results of different four networks are shown in Fig 8.

In contrast, ResNet101 performed relatively well compared to DenseNets, VGG16 and InceptionV4 as illustrated in Fig 8 and Table 2. For Fig 8, the DenseNets121 has the best performance in training set, but it have the worst performance in testing set. The VGG16 has poor performance in both training and testing sets. The reason is that the DenseNets121 is deeper network structure, the learning of image-features may be overfitting in training set, and then the accuracy rate is lowest in testing set. The VGG16 is simpler network structure, which cannot fully extract the high-level semantics of image-features, and then the accuracy rate is lower in testing set. Overall, ResNet performed well with the highest test_accuracy (0.9935) and lowest test_loss (0.024).

**Table 2:** The comparison results of CNN model and traditional identification model.

Networks	Train_accuracy	Train_Loss	Test_accuracy	Test_Loss
VGG16	0.983	0.085	0.9441	0.156
DenseNets121	0.998	0.009	0.9256	0.271
InceptionV4	1.00	0.00	0.9744	0.201
ResNet10	0.999	0.001	0.9935	0.024

### The traditional identification method

#### The result of traditional identification model

**Fig 9:**
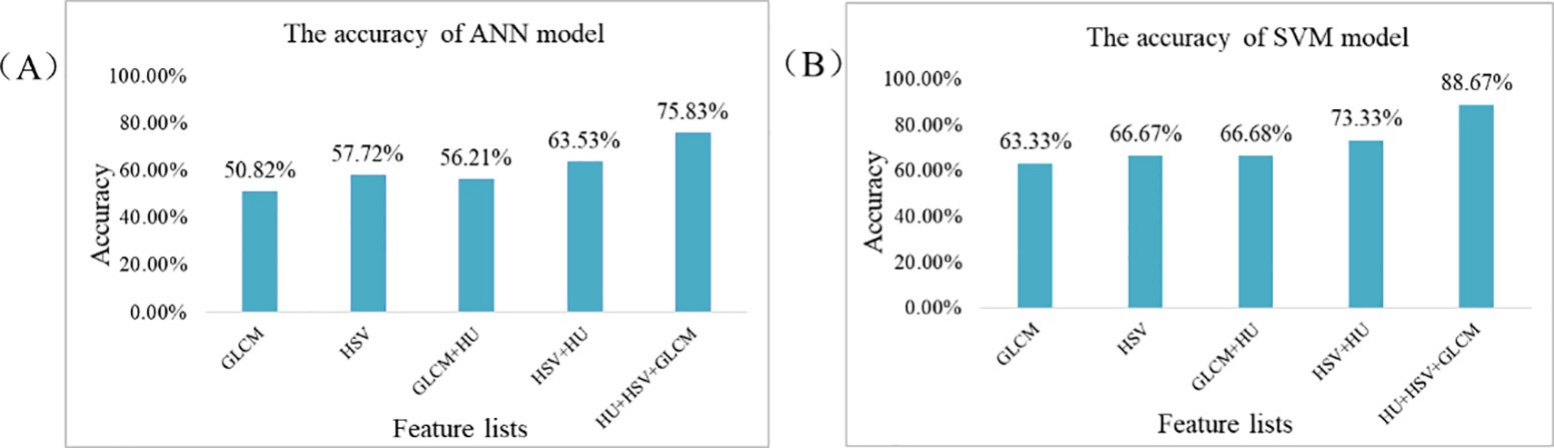
The different accuracy of multiple feature lists under BP and SVM model.

On the basic of ZPD, a total of 60 images of 300 samples in each type of ZP, which are selected as the data for traditional analysis algorithm. After data preprocessing, the features of color, shape and texture are obtained respectively. The BP and SVM algorithm are used to classify different species of ZP. The different identification result of multiple feature lists which based on the BP and SVM algorithm respectively, are shown in Fig 9.

As can be seen from the Fig 9, the accuracy of SVM model reported significantly higher than the BP model, and the SVM classifier is capable of distinguishing between poorly and highly informative features, since they target a solution that not only minimizes the training error, but also maximizes the margin distance between classes. It also can be found that the different accuracy is obtained from different feature lists. Based on the HU+HSV+GLCM, the highest accuracy is 88.67% in SVM. And the feature of color is most important for the identification of ZP among the three feature items. However, the limitation of this method is the influence of extracted features, when the environmental factors are not uniform or the background is complex. As the same time, the BP and SVM algorithms are used for restricted dataset. This method is not feasible for a large number of data. Thereby, it is a shortage for the actual applications.

#### The comparison of different methods

Using the preprocessed dataset, the comparison between the CNN model and the traditional identification model obtained with the SVM and BP, which use hand-crafted color, texture and shape features respectively. The performance of the proposed approach is evaluated to determine its generalization capability. And the comparison results are shown in Table 3.

**Table 3:** The comparison results of CNN model and traditional identification model.

Methods	Data	Accuracy	Missing
HSV+HU+GLCM+BP	300	0.7583	0.2417
HSV+HU+GLCM+SVM	300	0.8867	0.1133
VGG16	65000	0.9441	0.0559
ResNet	65000	0.9935	0.0065
InceptionV4	65000	0.9744	0.0256
DenseNets121	65000	0.9256	0.0744

According to the comparison results show that the effectiveness of CNN models have better performance than BP/SVM and ResNet model has the highest accuracy for the identification of ZP.

## Conclusion

In this study, a novel approach based on effective image detection and image identification is proposed to identify the different species of ZP. According to ZPD, firstly, the data considering 2 categories are trained to detect the labels by YOLO. Then the detection results are used to train the multiple deep learning including VGG, ResNet, Inception v4, and DenseNet. Experimental results demonstrate that ResNet have the highest identification accuracy is 99.35%. And compared with the traditional machine learning algorithms including BP and SVM, the proposed method has a better performance, faster convergence rate, as well as higher recognition ability. In the further, the success of this research will develop a new method for the fast and accurate classification of different species TCMs.

## Supporting information

S1 Table VGG16, ResNet101, Inception v4 and DenseNet121 respectively. (DOCX)

S2 Table The crucial parameters of network. (DOCX)

S3 Table The crucial parameters of network. (DOCX)

S4 Table The TPR and FPR metrics table. (DOCX)

S5 Table The crucial parameters of network. (DOCX)

S6 Table The crucial parameters of network. (DOCX)

S7 Table The TPR and FPR metrics table. (DOCX)

S1 Fig The traditional identification process. (DOCX)

S2 Fig The CNN architecture. (DOCX)

S3 Fig Flow charts of (A) training mode and (B) testing mode.
(DOCX)
