# Supplementary material for: Identification of different species of Zanthoxyli Pericarpium based on convolution neural network
Source: PLoS One. 2020 Apr 13;15(4):e0230287. doi: 10.1371/journal.pone.0230287 (PMC7153909; doi:10.1371/journal.pone.0230287)
Supplement: S1 Table — (DOCX) [file pone.0230287.s001.docx]

# S1 Table. The architecture parameters of VGG16, ResNet101, Inception v4 and DenseNet121 respectively.

| Layer name | Structure | Output size |
| --- | --- | --- |
| block1 | 3×3,64  2×2 max pool  stride 2 | 112×112 |
| block2 | 3×3,128  2×2 max pool  stride 2 | 56×56 |
| block3 | 3×3, 256  2×2 max pool  stride 2 | 28×28 |
| block4 | 3×3, 512  2×2 max pool  stride 2 | 14×14 |
| block5 | 3×3, 512  2×2 max pool  stride 2 | 7×7 |
| block6 | FC-4096  FC-5 | 1×1 |

| Layer name | Structure | Output size |
| --- | --- | --- |
| Conv1 | 7×7, 64  stride 2 | 112×112 |
| Conv2_x | 3x3 max pool  stride 2  1×1, 64  3×3, 64 ×3  1×1, 256 | 56×56 |
| Conv3_x | 1×1, 128  3×3, 128 ×4  1×1, 512 | 28×28 |
| Conv4_x | 1×1, 256  3×3, 256 ×23  1×1, 1024 | 14×14 |
| Conv5_x | 1×1, 512  3×3, 512 ×3  1×1, 2048 | 7×7 |
| Classification Layer | average pool  5-d fc  softmax | 1x1 |

| Layer name | Structure | Output size |
| --- | --- | --- |
| Stem | 3×3, 32, stride 2; 3×3, 64  3×3, max pool, stride 2; 3×3, 96, stride 2  1×1, 64; 7×1, 64; 3×3, 96  3×3, conv; max pool, stride 2 | 35×35 |
| 4*Inception-A | 1×1, 64; 3×3, 96  1×1, 64; 3×3, 96  1×1, 96  average pool, 1×1, 96 | 35×35 |
| Reduction-A | 1×1, conv; 3×3, conv; 3×3, conv, stride 2  3×3, conv  3×3, max pool, stride 2 | 17×17 |
| 7*Inception-B | 1×1, 192; 1×7, 192; 7×1, 224; 7×1, 256  1×1, 192; 1×7, 224; 1×7, 256  1×1, 384  average pool; 1×1, 128 | 17×17 |
| Reduction-B | 1×1, 256; 1×7, 256; 7×1, 320; 3×3, 320, stride 2  1×1, 192; 3×3, 192, stride 2  3×3, max pool, stride 2 | 8×8 |
| 3*Inception-C | 1×1, 384; 1×3, 448; 3×1, 512; 1×3, 256  1×1, 384; 1×3, 256; 3×1, 256  1×1, 256  average pool; 1×1, 256 | 8×8 |
| Classification Layer | average pool  Dropout  5-d fc,  softmax | 1×1 |

| Layer name | Structure | | Output size |
| --- | --- | --- | --- |
| Conv | 7×7, conv  stride 2 | | 112×112 |
| Pooling | 3×3, max pool  stride 2 | | 56×56 |
| Dense Block (1) | 1×1, conv 3×3, conv | ×6 | 56×56 |
| Transition Layer (1) | 1×1, conv | | 56×56 |
|  | 2×2, average pool  stride 2 | | 28×28 |
| Dense Block (2) | 1×1, conv 3×3, conv | ×12 | 28×28 |
| Transition Layer (2) | 1×1, conv | | 28×28 |
|  | 2×2, average pool  stride 2 | | 14×14 |
| Dense Block (3) | 1×1, conv  3×3, conv | ×24 | 14×14 |
| Transition Layer (3) | 1×1, conv | | 14×14 |
|  | 2×2, average pool  stride 2 | | 7×7 |
| Dense Block (4) | 1×1, conv  3×3, conv | ×16 | 7×7 |
| Classification Layer | 7×7, global average pool,  5-d fc,  softmax | | 1×1 |
