# Supplementary material for: Identification of different species of Zanthoxyli Pericarpium based on convolution neural network
Source: PLoS One. 2020 Apr 13;15(4):e0230287. doi: 10.1371/journal.pone.0230287 (PMC7153909; doi:10.1371/journal.pone.0230287)
Supplement: S2 Table — (DOCX) [file pone.0230287.s002.docx]

# S2 Table. The crucial parameters of Network

| Parameters | Value |
| --- | --- |
| momentum | 0.9 |
| weight decay | 0.0005 |
| max batches | 45000 |
| learning rate | 0.0001(0~100) |
|  | 0.001(100~25000) |
|  | 0.00001(25000~35000) |
|  | 0.00001(35000~45000) |
