# Supplementary material for: Identification of different species of Zanthoxyli Pericarpium based on convolution neural network
Source: PLoS One. 2020 Apr 13;15(4):e0230287. doi: 10.1371/journal.pone.0230287 (PMC7153909; doi:10.1371/journal.pone.0230287)
Supplement: S3 Table — (DOCX) [file pone.0230287.s003.docx]

# S3 Table. The crucial parameters of Network

| Parameters | Value |
| --- | --- |
| momentum | 0.9 |
| weight decay | 0.0005 |
| max_iter | 250000 |
| learning rate | 0.0001/0.001 |
| dropout | 0.2 |
