# Supplementary material for: Identification of different species of Zanthoxyli Pericarpium based on convolution neural network
Source: PLoS One. 2020 Apr 13;15(4):e0230287. doi: 10.1371/journal.pone.0230287 (PMC7153909; doi:10.1371/journal.pone.0230287)
Supplement: S4 Table — (DOCX) [file pone.0230287.s004.docx]

# S4 Table. The TPR and FPR metrics table

|  | Actual Positive result | Actual Negative result |
| --- | --- | --- |
| Prediction result (P) | TP | FP |
| Prediction result (N) | FN | TN |
