# Supplementary figures and images for: Identification of different species of Zanthoxyli Pericarpium based on convolution neural network
Source: PLoS One. 2020 Apr 13;15(4):e0230287. doi: 10.1371/journal.pone.0230287 (PMC7153909; doi:10.1371/journal.pone.0230287)

# Supporting information

# S1 Fig. The Traditional identification process


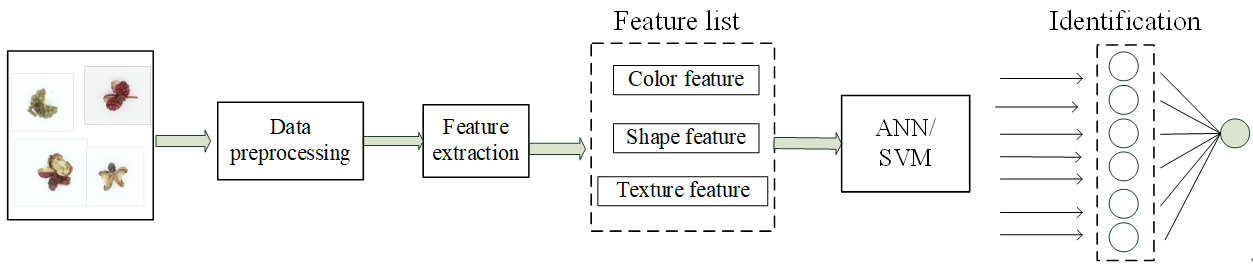

Supplement: S1 Fig — (DOCX) [file pone.0230287.s008.docx]

# Supporting information

# S2 Fig. The CNN architecture


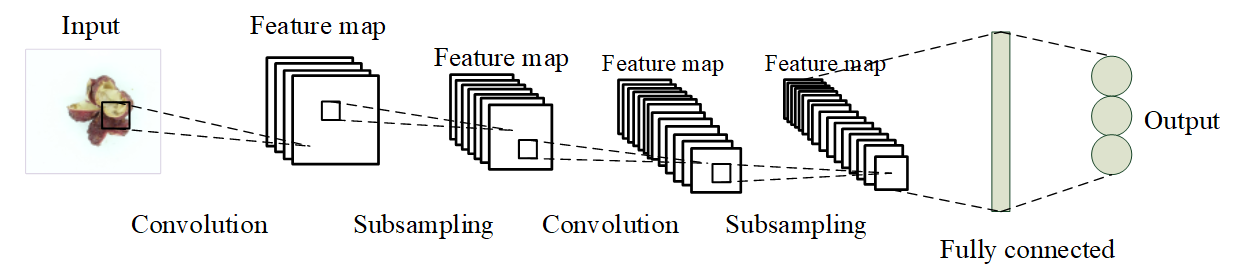

Supplement: S2 Fig — (DOCX) [file pone.0230287.s009.docx]

# Supporting information

# S3 Fig. Flow charts of (A) Training mode and (B) Testing mode


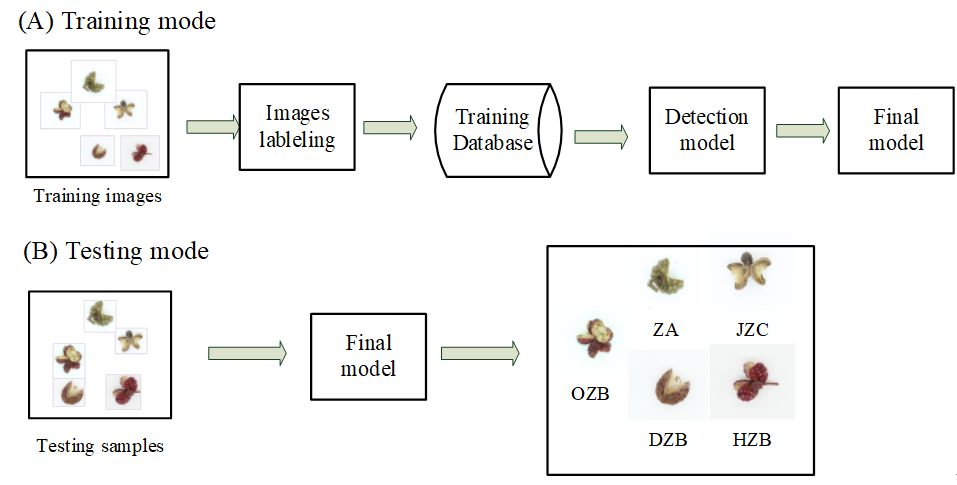

Supplement: S3 Fig — Flow charts of (A) training mode and (B) testing mode. (DOCX) [file pone.0230287.s010.docx]
